# Left atrial dilation in patients with heart failure and preserved ejection fraction: Value of CMR Simpson method

**DOI:** 10.1186/1532-429X-18-S1-P290

**Published:** 2016-01-27

**Authors:** Vassilis Vassiliou, Hitesh Patel, Carl Hayward, Gillian C Smith, Ricardo Wage, Stuart D Rosen, Alexander Lyon, Francisco Alpendurada, Dominique Auger, Dudley J Pennell, Carlo Di Mario, Sanjay Prasad

**Affiliations:** 1 grid.439338.6CMR, Royal Brompton Hospital, London, United Kingdom; 2 grid.7445.20000000121138111National Heart and Lung Institute, Imperial College London, London, United Kingdom

## Background

Left atrial (LA) dilation is an important prognostic indicator in heart failure. CMR provides the gold standard for LA volume estimation using the volumetric short axis Simpson method. However, this requires the acquisition of additional images with breatholding which can be difficult in patients with heart failure. We investigated whether volume calculated by the biplane area-length method from CMR and echocardiography were appropriate alternatives in a cohort of patients with heart failure and preserved ejection fraction (HFpEF).

## Methods

25 patients with HFpEF (age 74 ± 6 years, 15 male) underwent up to 3 CMR and 3 echocardiography studies each giving a total of 72 scans for each modality. CMR and echo were undertaken the same day. SSFP cine imaging with contiguous stack of short-axis images across the LA was taken to calculate LA volume using Simpson method. Standard 2 chamber and 4 chamber were also obtained to calculate LA volume using biplane area-length method from both CMR and echo. The biplane area-length method volume was calculated using the equation 8/3π*A1*A2*L where A1 and A2 were the areas in the 2 and 4 chamber and L was the shortest linear measurement parallel to the atrial septum in the 2 or 4 chamber view.

## Results

**Figure 1:**
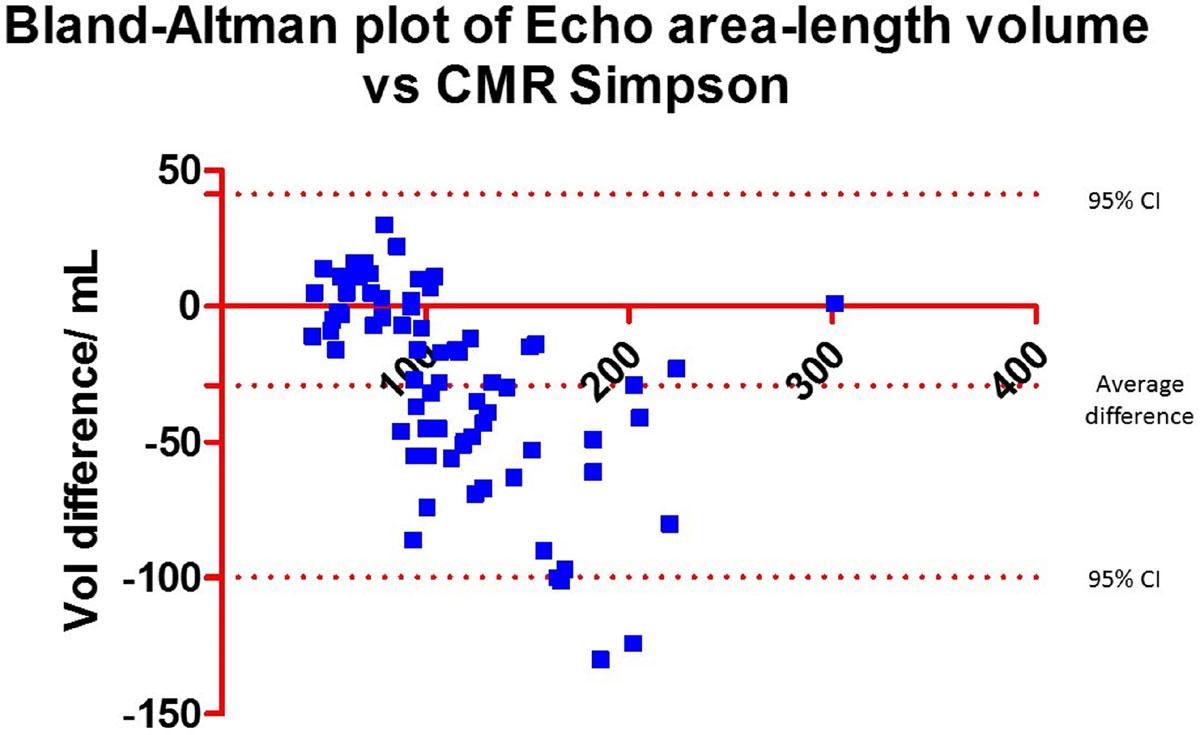
**Table on the left showing the LA volume as estimated using area-length method from CMR and echocardiography comparing these to the gold standard CMR Simpson method**. On the right, Bland-Altman of the Echo LA size compared to the CMR Simpson, showing that at normal volumes echocardiography performs well. However at higher LA volumes (>150 mls) echocardiography systematically underestimates compared to the gold standard.

72 studies were performed in total. 43 studies were performed during Atrial Fibrillation (AF), and 29 in sinus rhythm (SR). The absolute LA volumes using the three methods with the standard deviation are shown in Figure 1. Compared to the volumetric short axis technique, the echo area-length method underestimated LA volume in both SR (p=0.0009) and AF (p=<0.0001). CMR area-length showed a trend to overestimation of the LA volume in SR (p=0.053) but not in AF (p=0.927).

**Figure 2:**
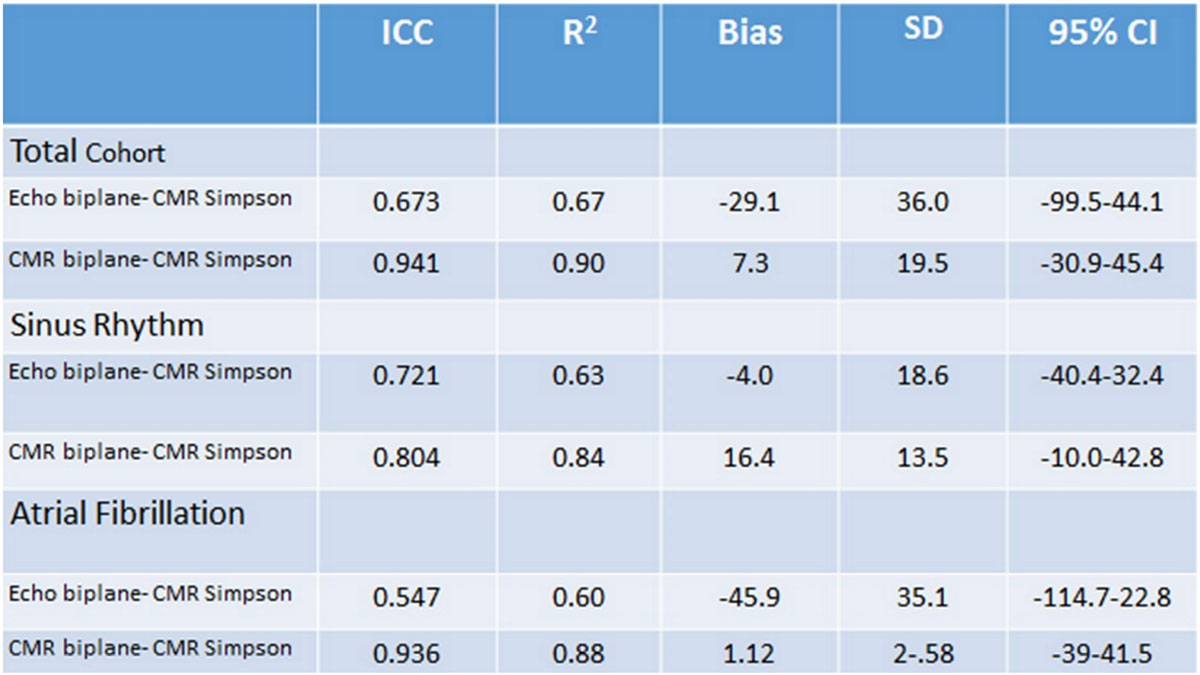
**Table showing the correlation between the three methods in the SR, AF and the combination of the two**. CMR area-length method provided an accurate alternative to the gold standard, whereas echocardiography was less reliable particularly in AF.

Intraclass correlation coefficients (ICC) and mean bias with 95% confidence intervals using the Bland-Altman plot method between the techniques are presented in the Tables in Figure 1 and 2. There was good agreement between LA volume derived from short axis stacks and the area-length method on CMR (ICC=0.94), and this relationship was preserved in patients with AF. Comparison of LA volume derived from echocardiography with the CMR short axis stack suggested good agreement when the LA volume was normal but a trend to systematic underestimation by echocardiography in patients with pathological left atrial enlargement (Figure 1). This trend was more pronounced in patients with AF.

## Conclusions

In a cohort of patients with HFpEF, CMR area-length method for estimating LA volume in both SR and AF correlated and agreed well with the gold standard CMR Simpson LA volumetric stack method. This suggests that in patients with HFpEF area-length volume calculation of LA is reliable and can be considered to shorten scan duration. Echocardiographic data, especially in AF, appeared less accurate.

